# Jwa Kum Whan Attenuates Nonalcoholic Fatty Liver Disease by Modulating Glucose Metabolism and the Insulin Signaling Pathway

**DOI:** 10.1155/2019/4589810

**Published:** 2019-02-10

**Authors:** Dong-Woo Lim, Hyuck Kim, Seung-Jun Lee, Ga-Ram Yu, Jai-Eun Kim, Won-Hwan Park

**Affiliations:** ^1^Department of Pathology, College of Korean Medicine, Dongguk University, Dongguk-ro 32, Goyang 10326, Republic of Korea; ^2^Department of Diagnostics, College of Korean Medicine, Dongguk University, Dongguk-ro 32, Goyang 10326, Republic of Korea; ^3^Institute of Korean Medicine, Dongguk University, Dongguk-ro 32, Goyang 10326, Republic of Korea

## Abstract

Over the last decade, the link between nonalcoholic fatty liver disease (NAFLD) and insulin resistance has attracted considerable attention. Caused by chronic hyperglycemic stress, insulin resistance (IR) impairs insulin signal transduction and leads to the development of NAFLD. Jwa Kum Whan (JKW), a herbal formula in Traditional Korean Medicine, consists of two medicinal herbs that possess notable effects against hyperglycemia and IR. In this study, we sought to determine the pharmacological effects of JKW, and the mechanisms responsible, on hepatic steatosis in free fatty acids (FFAs)-stimulated HepG2 cells and in high-fat diet (HFD)-fed obese mice. Treatment with JKW significantly decreased intracellular lipid accumulation* in vitro*. Furthermore, JKW significantly triggered the phosphorylation of insulin receptor substrate-1 (IRS-1) and phosphoinositide 3-kinase (PI3K) and modulated glucose and lipid metabolism via an AMP-activated protein kinase (AMPK) signaling pathway. Analysis of serum parameters in HFD-fed mice showed that JKW improved glucose levels and insulin resistance index (HOMA-IR). In addition, JKW successfully reduced hepatic triglyceride (TG) and cholesterol accumulation. Our results suggest that JKW alleviates NAFLD by modulating the insulin signaling pathway and glucose metabolism. These findings provide a scientific rationale for the potential use of JKW for the treatment and prevention of NAFLD.

## 1. Introduction

Nonalcoholic fatty liver disease (NAFLD) is the most common liver disease and is getting increasingly recognized as a global health burden in the emerging economies of Asia as well as in Western populations [1, 2]. NAFLD is characterized by wide spectrum of hepatic lipid accumulation, ranging from simple steatosis to nonalcoholic steatohepatitis (NASH) [3]. NASH is a unique entity characterized by fatty changes with lobular hepatitis in the absence of alcoholism [4]. Furthermore, NAFLD and NASH are intermediate and reversible disease stages with adjustable causal factors and preceding liver fibrosis and cirrhosis [5, 6] and are strongly associated with characteristics of metabolic syndrome, such as obesity, diabetes, dyslipidemia, and cardiovascular disease [7]. Thus, to prevent further progression of liver disease, it is important to control the underlying factors of metabolic syndrome.

Among these factors, type 2 diabetes (T2DM) has been frequently associated with NAFLD [8]. According to a population-based epidemiological study, about 70% of T2DM patients have NAFLD [9], and a cross-sectional study revealed impaired glucose metabolism is independently associated with NAFLD in Japanese adult population [10].

Insulin resistance (IR) is a pathological condition, in which glucose uptake, metabolism, and storage are not adequately controlled by insulin [11]. By intensive investigation on the link between NAFLD and IR over the last decade, IR has been identified as a major contributor to the development of NAFLD, as determined by the “two-hit” hypothesis [12, 13]. For IR is a major clinical marker of metabolic syndrome, which is closely connected to T2DM [14, 15], some antidiabetic agents directly or indirectly ameliorate IR. [16]. Accordingly, medications for treating glucose abnormalities offer feasible strategies for alleviating NAFLD and NASH through modulation of IR.

Jwa Kum Whan (JKW) is a traditional herbal prescription used to treat digestive disorders, such as chronic gastritis and hyperacidity, composed of the roots of* Scutellaria baicalensis* and fruits of* Evodia rutaecarpa *[17]. It has been reported that* S. baicalensis, *the major constituent of JKW, exhibits pharmacological activities and possible therapeutic effects in liver of diabetic mice (db/db) and in human hepatocellular carcinoma [18], and, in another study, it was found to alleviate IR by improving insulin sensitivity [19]. Similarly, it has been reported that evodiamine, a major component of* E. rutaecarpa*, inhibited insulin resistance and improved glucose tolerance in an obese/diabetic mouse model [20]. Furthermore, two active alkaloids found in* E. rutaecarpa* were reported to have beneficial pharmacological effects on metabolic syndrome [21]. Based on these favorable effects and mechanism on glucose metabolism, we assumed that JKW attenuates NAFLD in obese mice.

In the present study, we evaluated the ameliorating effects of JKW on NAFLD in high-fat diet (HFD)-fed mice and on free fatty acids (FFAs)-induced lipid accumulation in human hepatocytes. In addition, we investigated the biomolecular mechanisms responsible for the effects of JKW on glucose metabolism and the insulin signaling pathway and, thus, on NAFLD.

## 2. Materials and Methods

### 2.1. Preparation of JKW Extract

*Evodiae Fructus* and* Scutellariae Radix *were purchased as dried herbs from Human herb (Gyeongsangbuk-do, Republic of Korea) and authenticated by the corresponding author. Voucher specimens were deposited at the Institute of Korean Medicine, Dongguk University (No. 2017-DGMRC-081). Extracts were mixed in the ratio described in Donguibogam, a classic text on Korean medicine [22]. In brief, 240 g of Scutellariae Radix and 40 g of Evodiae Fructus were ground, boiled in distilled water for 3 h, filtered through Whatman 3 filter paper, and concentrated by vacuum evaporation (Buchi, Flawil, Switzerland). The dried powder obtained was freeze dried for 3 days using a freeze-dryer system (FD1100, EYELA, Japan), and the herbal formula produced was stored at -80°C until required (yield 20.27%).

### 2.2. Animal Experiment.

Twenty-four C57BL/6J mice, each weighing 18-19 g, were provided by OrientBio (Gyeonggi-do, Republic of Korea) and bred in the Animal Center of Dongguk University of Pharmacy. Mice were housed under specific pathogen-free conditions at 24 ± 2°C and 40 ± 1% humidity under 12 h dark-light cycle with free access to purified water and standard diet. After a week of acclimatization, mice were divided randomly into four groups (6 mice per group): normal diet group, HFD group (60% HFD), low-dose JKW (HFD with 100 mg/kg body weight), and high-dose JKW (HFD with 200 mg/kg body weight). Mice were orally fed JKW once daily for 15 weeks and subjected to oral glucose tolerance test. Mice were then starved for 12 h, euthanized, and organs and blood were collected. Samples of organs were stored at -80°C for further study. The serum was separated from blood by centrifugation at 3000 rpm for 20 min and stored at 4°C for biochemical analysis. All animal experimental protocols were approved by the Animal Ethical Committee of Dongguk University (IACUC-2016-055-1).

### 2.3. Cell Culture and Viability Assay

HepG2 cells (a human hepatocellular carcinoma cell line) were purchased from the Korean Cell Line Bank (no. 88065, Seoul, Republic of Korea) and cultured in Dulbecco's Modified Eagles Medium (DMEM, Hyclone, Logan, UT, USA) supplemented with 10% FBS (Invitrogen, Grand Island, NY, USA) and a penicillin/streptomycin mix (Invitrogen) in a humidified 5% CO_2_ atmosphere at 37°C. The effect of JKW on cell viability was assessed using tetrazolium-based EZ-cytox kit (Daeil Lab, Seoul, Republic of Korea). In brief, cells were seeded at 1 × 10^4^ cells per well in 96-well plates and starved for 24 h before treatment. Cells were treated with different concentrations of JKW and incubated for another 24 h. Optical density of supernatants were measured at 450 nm using a microplate reader (VersaMax, Molecular Devices, CA, USA).

### 2.4. Measurement of Lipid Accumulation

HepG2 cells were seeded at 2 × 10^5^ cells per well in 24-well plates and starved for 24 h before treatment. Intracellular lipid accumulation was induced by adding a mixture of free fatty acids (FFAs; oleic acid and palmitic acid (2:1, v/v, respectively) dissolved in DMSO, 1 mM of final concentration) to serum-free DMEM containing 1% bovine serum albumin (BSA) for 24 h. Intracellular triglycerides (TG) and total cholesterol (TC) were assessed as previously described with slight modification [23]. In brief, supernatants were removed and cells were washed twice with Dulbecco's phosphate buffered saline (DPBS, Hyclone), supplemented with fresh culture media and treated with JKW(10 or 25 *μ*g/mL) for 48 h. Cells were then harvested, centrifuged at 12,000 rpm for 1 min, and the pellets were sonicated to obtain lysates for TG and TC analysis. Lipid levels were determined using a commercial kit (Asan Pharmacy, Seoul, Republic of Korea) by colorimetric analysis using standard protocols.

Lipid accumulation was assessed by Oil Red O staining. In brief, cells were prepared as described above, fixed with 10% formalin at room temperature, washed with 60% isopropanol, air-dried, stained with Oil Red O for 15 mins, and washed three times with distilled water. Images were captured using an inverted microscope and camera system (DMI 6000, Leica, Germany). Oil Red O was redissolved in pure isopropanol for 10 min and optical densities were measured using a microplate reader at 520 nm.

### 2.5. Glucose Uptake Monitoring

Glucose uptake was evaluated using a fluorescence assay. HepG2 cells were plated at 1 × 10^4^ cells per well in 96 well plates and incubated for 24 h. Cells were then starved for 24 h plated in serum-free DMEM containing palmitate (250 *μ*M) and treated with various concentrations of JKW (0 - 50 *μ*g/mL) for 24 h. Supernatants were then removed, cells were gently washed with DPBS, and media were replaced with glucose-free DMEM containing 2-NBDG (150 *μ*g/mL). Cells were then incubated at 37°C for 20 min and rewashed with DPBS. Relative fluorescences were obtained at excitation and emission wavelengths of 485 nm and 545 nm, respectively, using a fluorescence microplate reader (Spectra Gemini, Molecular Devices). Fluorescence images were obtained using an Olympus BX50 fluorescence microscope (Olympus, Tokyo, Japan).

### 2.6. Western Blot Analysis

HepG2 cells were lysed with RIPA buffer containing a protease and phosphatase inhibitor cocktail (Gendepot, Baker, TX, USA). Protein concentrations were determined using the BCA protein assay kit (Thermo Scientific, Waltham, MA, USA). In brief, 30 *μ*g of protein mixed with Lane Marker Reducing sample buffer (Thermo Scientific) was loaded onto SDS-PAGE gel and transferred to polyvinylidene difluoride membranes (GE Healthcare, Buckinghamshire, BK). Membranes were blocked with 5% BSA in Tris-buffered saline containing 0.1% Tween (TBST) for 1 h and incubated overnight at 4°C with primary antibodies against glucose transporter 2 (Glut2; Santa Cruz Biotechnology, Inc., Santa Cruz, CA, USA), AMP-activated protein kinase (AMPK; Cell Signaling Technology, Inc., Danvers, MA, USA), peroxisome proliferator-activated protein gamma (PPAR*γ*; Cell Signaling Technology), CCAAT/enhancer-binding protein alpha (C/EBP*α*; Cell Signaling Technology), carnitine palmitoyltransferase-1 (CPT-1; Cell Signaling Technology), insulin receptor substrate-1 (IRS-1; Santa Cruz Biotechnology), phosphoinositide 3-kinase (PI3K; Cell Signaling Technology), protein kinase B (AKT; Cell Signaling Technology), *β*-actin (Santa Cruz Biotechnology), and the phosphorylated forms of AMPK (Thr 172), IRS-1 (Ser 612), PI3K (Tyr 458), and AKT (Ser 473) (1:1000 dilution in TBST containing 3% BSA, Santa Cruz Biotechnology). After incubation, membranes were washed with TBST three times and further incubated with horseradish peroxidase (HRP)-conjugated secondary antibodies (1:2000 dilutions in TBST containing 1% BSA, Santa Cruz Biotechnology) for 2 h. Blots were detected using a western blot imaging system (Fusion Solo, Vilber Lourmat, Collégien, France) equipped with analysis software using *β*-actin, as a loading control.

### 2.7. Fasting Glucose and HOMA-IR Analysis

In the final week of animal experiment, mice were starved for 12 h before conducting the glucose tolerance test. In brief, a 2 g/kg of bodyweight of D-glucose (dissolved in sterile distilled water) was administered orally and glucose levels in tail vein blood were determined at 0, 30, 60, 90, and 120 min later using a glucometer and strip (ACCU-CHEK, Roche, Basel, Switzerland). HOMA-IR indices, which are measures of insulin resistance, were calculated using

(1) HOMA-IR=fasting  glucose×fasting  insulinmU/L22.5

### 2.8. Serum Biochemistry and Inflammatory Cytokines

Blood serum analyses including serum TG, cholesterol, high-density lipoprotein (HDL), glutamic oxaloacetic transaminase (GOT), and glutamic pyruvic transaminase (GPT) levels were performed using commercial colorimetric kits (Asan Pharmacy). Relative absorbances were recorded at 550 nm (serum TG), 500 nm (cholesterol or HDL), and 505 nm (GOT or GPT) using a microplate reader. Serum insulin levels were measured using a commercial mouse insulin ELISA kit (Mercodia AB, Uppsala, Sweden). Inflammatory cytokines including interleukin-6 (IL-6) and interleukin-beta (IL-*β*) levels were measured using a mouse-specific ELISA kit (Thermo Scientific) at 450 nm.

### 2.9. Liver Tissue Analysis

Tissue samples obtained from same liver sections were sliced, homogenized, and centrifuged. Supernatant was subjected to lipid content analysis. Hepatic TG and cholesterol levels were determined using a tissue-specific analysis kit (Asan Pharmacy). Individual results were normalized versus total protein concentration. Lipid peroxidation was assessed as previously described with slight modification [23]. In brief, equal amounts of liver homogenates were mixed with 0.81% 2-thiobarbituric acid (w/v), heated at 95°C for 1 h, and mixed with 1 mL of distilled water and 5 mL of pyridine/1-butanol mixture (1:15, v/v). Reactants were centrifuged at 3000 rpm for 30 min and lipid peroxidation levels determined by measuring absorbance at 532 nm.

### 2.10. Statistical Analysis

Results are presented as the means ± standard deviations (SDs) of at least three independent experiments. The analysis was conducted using the Student's* t*-test in SigmaStat 3.5 (Systat Software Inc., CA, USA). Statistical significance was accepted for p values < 0.05.

## 3. Results

### 3.1. JKW Attenuated Lipid Accumulation in FFAs-Stimulated HepG2 Cells

**Figure 1:**
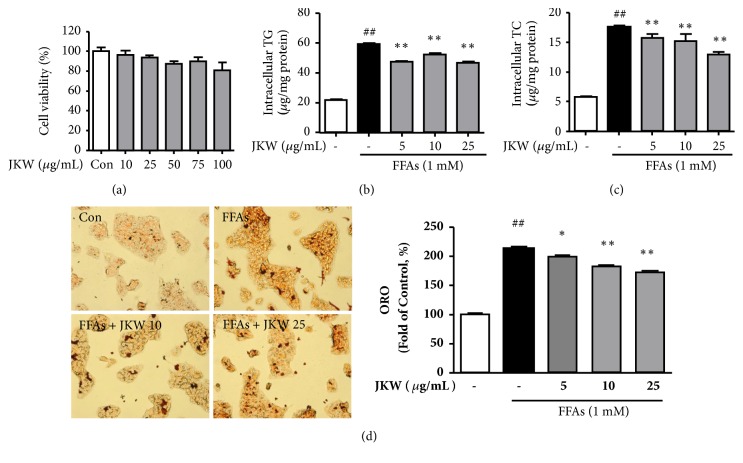
Effects of JKW on intracellular TG and TC and on lipid accumulation in HepG2 cells. (a) Cell viability was determined using the MTT-based Ez-cytox assay. Cells were treated with different concentrations of JKW extract (10, 25, 50, 75, or 100 *μ*g/mL) for 24 h. Results are expressed as percentages of untreated controls. (b) Intracellular TG and (c) TC contents were measured as described in Materials and Methods. (d) Lipid accumulation was determined using an Oil Red O assay. Results are the means ± SDs of three independent experiments. ^##^*p* < 0.01 vs. untreated controls and ^*∗∗*^*p* < 0.01 vs. FFA-treated controls.

JKW at concentrations of < 25 *μ*g/mL had insignificant effects on HepG2 cell viability (Figure 1(a)). We then investigated the effects of JKW on intracellular lipid contents in FFAs induced steatosis cells. FFAs treatment increased lipid accumulation, but JKW treatment significantly reduced intracellular TG (Figure 1(b)) and TC (Figure 1(c)) levels. In addition, Oil Red O staining showed JKW posttreatment also significantly reduced lipid accumulation in HepG2 cells (Figure 1(d)).

### 3.2. JKW Improved Glucose Utilization in FFAs-Stimulated HepG2 Cells

**Figure 2:**
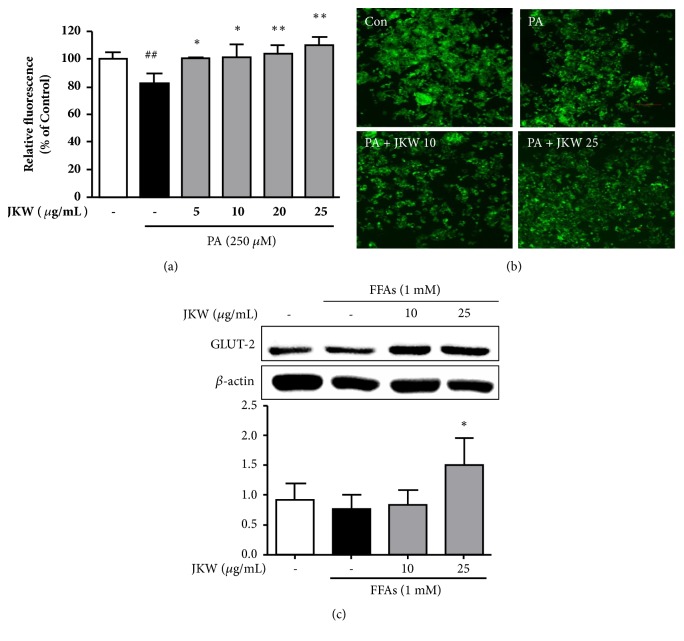
Effects of JKW on glucose metabolism in HepG2 cells. (a) After 24 h of starvation, cells were cotreated with palmitate (250 *μ*M) and JKW (5, 10, 20, or 25 *μ*g/mL) for 24 h. Glucose uptakes were assessed based on the amount of 2-NBDG absorbed by cells as determined by measuring relative fluorescence intensities. (b) Fluorescence images were obtained by fluorescence microscopy. (c) GLUT-2 levels were assessed by Western blot and results are quoted as relative protein expressions normalized versus *β*-actin, which was used as a loading control. Results are the means ± SDs of three independent experiments. ^##^*p* < 0.01 vs. untreated controls. ^*∗*^*p* < 0.05 and ^*∗∗*^*p* < 0.01 versus FFA-treated controls.

We analyzed the effect of JKW on glucose uptake by palmitic acid-stimulated HepG2 cells using fluorescence-labeled glucose. Relative fluorescence intensities markedly declined after treating cells with 250 *μ*M palmitic acid, indicating a substantial reduction in glucose metabolism (Figure 2(a)). But JKW cotreatment significantly elevated fluorescence intensities in dose-dependent manner. Microscopic observations showed JKW cotreatment increased fluorescence intensities, indicating recovery of glucose uptake (Figure 2(b)). Immunoblot results also showed palmitic acid-induced downregulations of GLUT2 in cytoplasm were recovered dose dependently by JKW (Figure 2(c)).

### 3.3. JKW Restored Insulin Signaling and Modulated Energy Metabolism in FFAs-Stimulated HepG2 Cells

**Figure 3:**
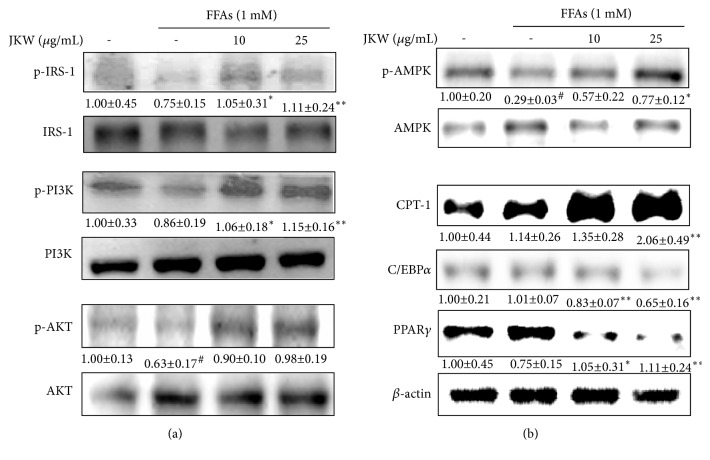
Effects of JKW on proteins related to insulin signaling and energy metabolism in HepG2 cells. (a) After starving HepG2 cells for 24 h, they were cotreated with FFAs (1 mM) and JKW (10 or 25 *μ*g/mL) for 24 h. Cells were incubated with 50 nM of insulin for 30 min before harvest. Relative IRS-1, PI3K, and AKT levels, which represent insulin signaling pathways, were assessed by dividing the expressions of phosphorylated proteins by those of corresponding nonphosphorylated proteins. (b) Relative levels of energy metabolism-related proteins (AMPK, CPT-1, C/EBP*α*, and PPAR*γ*) were determined by normalizing protein expressions versus *β*-actin protein (or phosphorylated AMPK) expression. Results are expressed as the means ± SDs of three independent experiments. ^#^*p* < 0.05 versus untreated controls. ^*∗*^*p* < 0.05 and ^*∗∗*^*p* < 0.01 versus FFA-treated controls.

Immunoblotting showed JKW activated insulin signaling via IRS-1, PI3K, and AKT after insulin stimulation. Levels of phosphorylated IRS-1 and PI3K were significantly and dose dependently increased by JKW treatment (Figure 3(a)). Furthermore, JKW at 10 or 25 *μ*g/mL increased AMPK phosphorylation by 96% and 168%, respectively (Figure 3(b)) and activated fatty acid oxidation by regulating CPT-1. In addition, the protein levels of adipogenic transcriptional factors, including C/EBP*α* and PPAR*γ*, were markedly diminished in HepG2 cells by JKW.

### 3.4. JKW Alleviated Glucose Parameters and Insulin Resistance in HFD-Fed Mice

**Figure 4:**
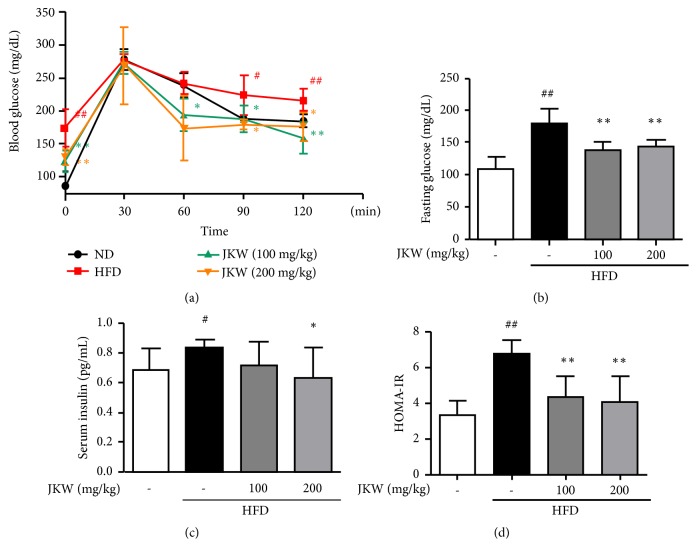
Effects of JKW on OGTT, fasting glucose, serum insulin, and HOMA-IR indices in mice fed on the HFD. (a) Impact of JKW on blood glucose levels as determined by OGTT at the indicated times after glucose loading. (b) Fasting glucose and (c) serum insulin levels were determined in mice fed on HFD as described in Materials and Methods. (d) HOMA-IR indices were used to determine insulin resistance in JKW-treated mice and these were compared with those of HFD controls. Results represent means ± SDs (n=6). ^#^*p* < 0.05 and ^##^*p* < 0.01 versus the normal diet group. ^*∗*^*p* < 0.05 and ^*∗∗*^*p* < 0.01 versus the HFD-fed group.

Oral glucose tolerance test (OGTT) results showed poor responses in HFD-fed mice to a heavy glucose load (Figure 4(a)). However, JKW stabilized blood glucose levels. The results obtained showed that JKW gradually improved glucose levels after 60 mins of glucose load and that this improvement was significant at 90 and 120 min in both low and high-dose JKW groups. Similarly, fasting glucose levels were significantly reduced in both JKW groups (Figure 4(b)). Furthermore, fasting insulin levels were reduced by JKW and reduction was significant in the 200 mg/kg group (Figure 4(c)). In addition, the calculated HOMA-IR indices were lower in the JKW-treated groups than in the HFD group (Figure 4(d)).

### 3.5. JKW Improved Serum Lipid Levels and Vital Hepatic Parameters in HFD-Fed Mice

**Figure 5:**
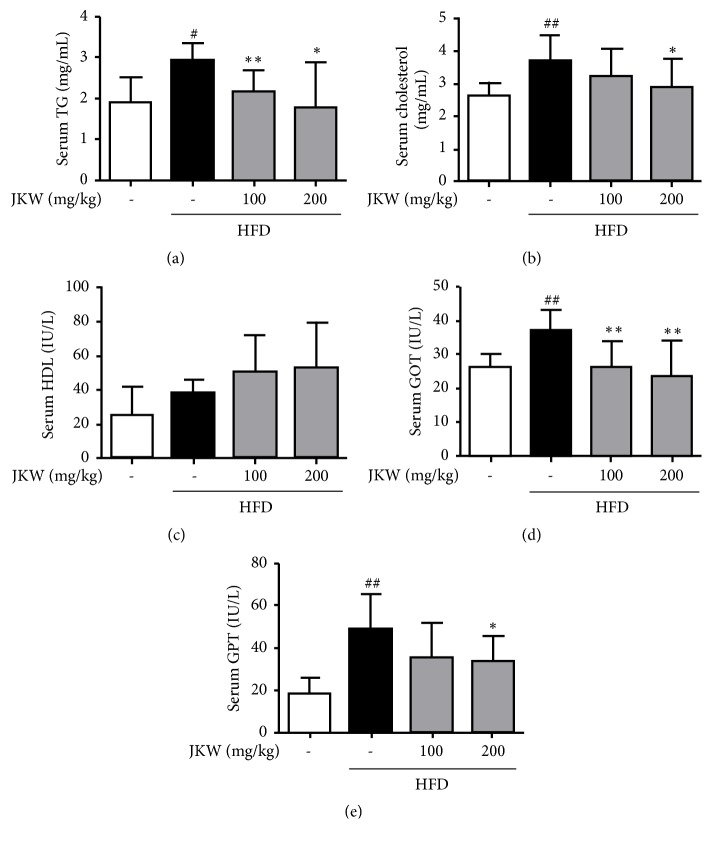
Effects of JKW on serum biochemical parameters in mice fed on the HFD. (a) Serum TG, (b) serum TC, and (c) high-density lipoprotein (HDL) levels were measured as described in Materials and Methods. (d) Serum GOT and (e) serum GPT levels were measured using colorimetric assay kits. Results represent means ± SDs (n=6). ^#^*p* < 0.05 and ^##^*p* < 0.01 versus the normal diet group. ^*∗*^*p* < 0.05 and ^*∗∗*^*p* < 0.01 versus the HFD-fed group.

**Figure 6:**
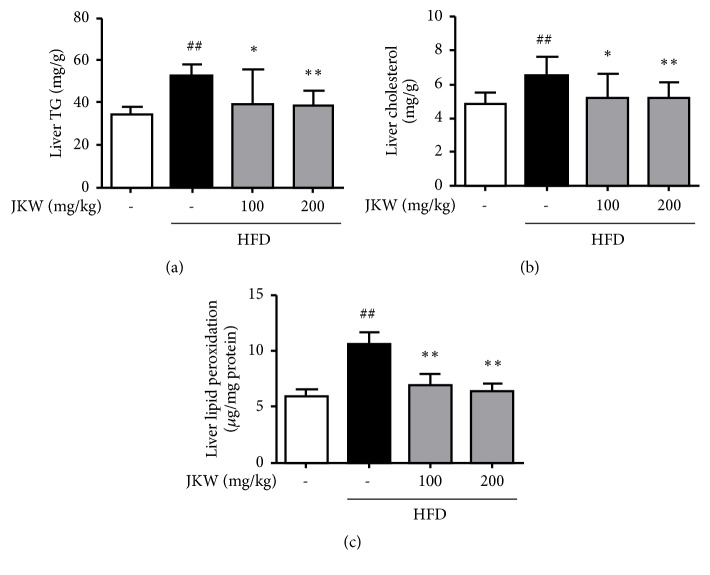
Effects of JKW on hepatic lipid profiles and oxidized lipid contents in mice fed on the HFD. (a) Liver TG and liver TC contents were measured using tissue-specific colorimetric assay kits. (c) Oxidized lipid contents were determined using a MDA-based assay as described in Materials and Methods. Results represent means ± SDs (n=6). ^##^*p* < 0.01 versus the normal diet group. ^*∗*^*p* < 0.05 and ^*∗∗*^*p* < 0.01 versus the HFD-fed group.

Hepatic fat deposits, liver and serum levels of TG and TC, oxidized hepatic lipids, and hepatic GOT and GPT levels in mice fed on the HFD showed metabolic features similar to human obesity [24, 25]. Results showed JKW significantly reduced all these variables in HFD-fed mice (Figures 5(a), 5(b), 5(d), 5(e), and Figures 6(a) and 6(b)). On the other hand, serum HDL was only increased lightly by JKW versus that observed in HFD-fed mice (Figure 5(c)). As shown in Figure 6(c), JKW administration caused a significant decline in hepatic oxidized lipid contents as compared with that observed in HFD-fed mice.

### 3.6. JKW Reduced HFD-Induced Low-Grade Chronic Inflammatory Cytokine Levels in HFD-Fed Mice

**Figure 7:**
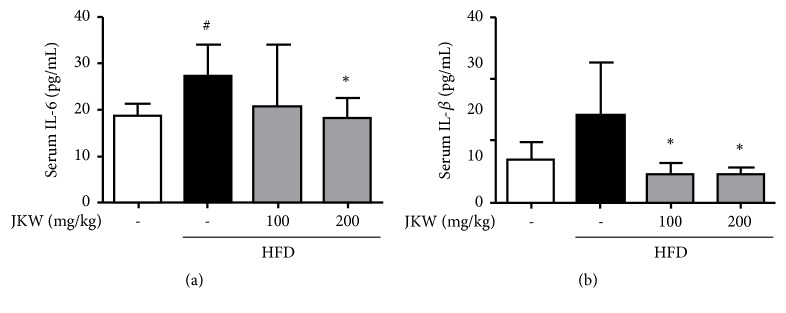
Effects of JKW on proinflammatory cytokine levels in mice fed on the HFD. (a) IL-6 and (b) IL-1*β* levels were measured using serum-specific colorimetric assay kits as described in Materials and Methods. Results represent means ± SDs (n=6). ^#^*p* < 0.05 versus the normal diet group and ^*∗*^*p* < 0.05 versus the HFD-fed group.

IL-6 levels were increased significantly and IL-1*β* levels nonsignificantly (due to considerable variation) by the HFD, and JKW treatment dose dependently reduced these increases (shown in Figure 7).

## 4. Discussion

Impaired glucose metabolism in diabetic patients has serious deleterious effects on diverse organs and tissues [22]. However, liver is the primary organ exposed to oxidative stress and is subjected to inflammatory cascades induced by hyperglycemia [26], and IR is a consequence of harmful oxidative stress [27] or inflammation [28]. Furthermore, because hyperglycemia diminishes normal cell functions, it affects the metabolisms of carbohydrates, proteins, and lipids in hepatic tissues.

Insulin resistance plays a key role in the progression from NAFLD to NASH or advanced liver disease [12, 13] and, thus, is viewed as a therapeutic target in NAFLD. Moreover, impaired intracellular insulin signaling pathways and increased HOMA-IR indexes are accepted markers of insulin resistance, which leads to abnormal blood lipid profiles and Very Low Density Lipoprotein 1 (VLDL1) increases [29, 30].

Treating hepatocytes with high concentrations of mixed FFAs is used to produce NAFLD models that resemble fatty liver disease in patients [31], and long-term feed supplementation with a HFD has been reported to result in NAFLD and human characteristics of obesity, insulin resistance, inflammation, and fibrosis in c57BL6J mice [25]. For these reasons, these two models are commonly adopted to investigate the likely impacts of unknown drugs against NAFLD* in vitro* and* in vivo*.

In the present study, JKW improved hyperglycemic status while attenuating insulin resistance in HFD-fed obese mice, and its favorable effects on glucose metabolism are well demonstrated by our* in vitro* and* in vivo *results. Glucose transporter 2 (GLUT-2) is a transmembrane carrier protein as it facilitates glucose transport [13], and our findings indicate JKW attenuated insulin resistance, and, thus, facilitated glucose utilization in our murine model. These effects were supported by increased 2-NBDG glucose uptake, elevated GLUT-2 protein expression, and activations of insulin pathways, as evidence by phosphorylated IRS-1 and phosphorylated PI3K levels. Furthermore, JKW was found to suppress serum glucose levels, serum insulin levels, and HOMA-IR indices* in vivo*.

The induction of AMPK phosphorylation by JKW suggests activation of lipid catabolism and the inhibition of lipogenesis was achieved via the modulations of downstream proteins [32]. It has been established that CPT-1 facilitates fatty acid transportation into mitochondria and, thus, promotes beta-oxidation [33], and C/EBP*α* and PPAR*γ* are essential transcription factors that participate in lipogenesis and increase hepatic triglyceride levels [34, 35], which we found were significantly modulated by JKW. These mechanisms may partially explain the reduction in intracellular lipid levels observed in our* in vitro* model.

Excessive levels of fatty acids in liver induce endoplasmic reticulum stress (ER stress), which in turn leads to oxidative stress and inflammation [36], which are believed as underlying mechanisms of NAFLD and NASH [27]. In the present study, 15 weeks of JKW administration significantly reduced lipid peroxidation and triglyceride and cholesterol accumulations in mice liver. The antioxidant activities of S. baicalensis and E. rutaecarpa [28, 37] might also contribute to the observed effects of JKW. In addition, JKW supplementation reduced hepatic damage caused by lipid accumulation and enhanced the activities of the liver enzymes GOT and GPT.

Proinflammatory cytokines cause insulin resistance in various tissues by hindering insulin signal transduction [38], and elevated levels of serum inflammatory cytokines, such as interleukin-6 (IL-6) and tumor necrosis factor *α* (TNF-*α*), have been reported in numerous* in vivo* and clinical studies conducted on diabetes [39, 40]. These cytokines exacerbate glucose homeostasis imbalance by impairing insulin production in pancreas [41] or impairing insulin signaling by decreasing the expressions of IRS-1 and GLUT4 [42, 43]. However, in the present study, JKW alleviated low grade inflammation and reduced inflammatory cytokine levels in HFD mice. These observations suggest the anti-inflammatory effects of JKW might help restore glucose metabolism in patients with NAFLD.

Our findings suggest JKW ameliorated NAFLD by controlling insulin signaling and glucose metabolism and by acting as an antioxidant and anti-inflammatory agent. Further study should be focused on identifying the compounds present in JKW, evaluating their efficacies and standardizing the herbal formula with appropriate quality control. In addition, we suggest the promising effects of JKW on metabolic syndrome observed in the present study be further investigated* in vitro* and* in vivo*.

## 5. Conclusion

JKW attenuated FFAs and HFD-induced NAFLD and had significant impacts on lipid accumulation and glucose-related indices* in vitro* and* in vivo*. In addition, JKW appeared to fundamentally improve insulin resistance and to activate AMPK and its downstream signals. Our findings suggest that JKW alleviate NAFLD by normalizing insulin signaling and glucose metabolism and that JKW be regarded a potential therapeutic candidate for the treatment of NAFLD.

## Data Availability

The data used to support the findings of this study are included within the article.
